# Evolutionary patterns of amino acid substitutions in 12 *Drosophila* genomes

**DOI:** 10.1186/1471-2164-11-S4-S10

**Published:** 2010-12-02

**Authors:** Lev Y Yampolsky, Michael A Bouzinier

**Affiliations:** 1 Department of Biological sciences, East Tennessee State University, Johnson City, TN 37614, USA; 2 InterSystems Corporation, One Memorial Drive, Cambridge, MA 02142, USA

## Abstract

**Background:**

Harnessing vast amounts of genomic data in phylogenetic context stemming from massive sequencing of multiple closely related genomes requires new tools and approaches. We present a tool for the genome-wide analysis of frequencies and patterns of amino acid substitutions in multiple alignments of genes’ coding regions, and a database of amino acid substitutions in the phylogeny of 12 *Drosophila* genomes. We illustrate the use of these resources to address three types of evolutionary genomics questions: about fluxes in amino acid composition in proteins, about asymmetries in amino acid substitutions and about patterns of molecular evolution in duplicated genes.

**Results:**

We demonstrate that amino acid composition of *Drosophila* proteins underwent a significant shift over the last 70 million years encompassed by the studied phylogeny, with less common amino acids (Cys, Met, His) increasing in frequency and more common ones (Ala, Leu, Glu) becoming less frequent. These fluxes are strongly correlated with polarity of source and destination amino acids, resulting in overall systematic decrease of mean polarity of amino acids found in *Drosophila* proteins. Frequency and radicality of amino acid substitutions are higher in paralogs than in orthologous single-copy genes and are higher in gene families with paralogs than in gene families without surviving duplications. Rate and radicality of substitutions, as expected, are negatively correlated with overall level and uniformity of gene expression. However, these correlations are not observed for substitutions occurring in duplicated genes, indicating a different selective constraint on the evolution of paralogous sequences. Clades resulting from duplications show a marked asymmetry in rate and radicality of amino acid substitutions, possibly a signal of widespread neofunctionalization. These patterns differ among protein families of different functionality, with genes coding for RNA-binding proteins differing from most other functional groups in terms of amino acid substitution patterns in duplicated and single-copy genes.

**Conclusions:**

We demonstrate that deep phylogenetic analysis of amino acid substitutions can reveal interesting genome-wide patterns. Amino acid composition of drosophilid proteins is shaped by fluxes similar to those previously observed in prokaryotic, yeast and mammalian genomes, indicating globally present patterns. Increased frequency and radicality of amino acid substitutions in duplicated genes and the presence of asymmetry of these parameters between paralogous clades indicate widespread neofunctionalization among paralogs as the mechanism of duplication retention.

## Background

Until recently, evolutionary genomics questions, including questions about amino acid composition of proteins, patterns of stabilising and positive selection and mechanisms of retention of duplicated genes and new function evolution, were typically answered either by analyzing phylogenies of select gene families [1,2] or by full-genome analysis of triplets of genomes with two ingroup genomes compared to measure evolutionary rates, while the third, outgroup, genome used to polarize the observed changes [3]. As the strategy of genome sequencing shifts from broad taxonomic coverage to sequencing multiple closely related genomes [4], a need arises in a set of tools to accomplish a phylogenetic analysis of amino acid substitutions in coding portions of a large number of protein families simultaneously and to address the question of generality of patterns observed in limited and possibly biased set of select gene families. Questions that can be asked using such approach include, but certainly are not limited to enquiries about long-term changes in amino acid compositions of proteins, about selective constrains and pressures across the genome and evolution of novel gene functions by retention and modification of duplicated genes. Here we present a tool to accomplish phylogenetic analysis of amino acids substitutions on the whole-genome scale using multiple amino acid alignments of over 11,000 gene families from twelve completely sequenced *Drosophila* genomes and illustrate its utility by the analysis of the resulting database of amino acid substitutions spanning 70 million years of drosophilid proteins evolution.

Global patterns of amino acid compositions of proteins is thought to not be at a detailed balance, but rather appears to be gradually evolving by consistently adding rare to and removing common amino acids from the amino acid repertoire of protein sequences [3,5]. There is an on-going debate on whether is pattern reflects the order in which amino acids have been added to the genetic code [3,6] or is caused by biases in mutability of particular codons [7-9]. As pointed out by [8], one way to address this controversy is to analyze the observed trends in a range of genomes of increasing degree of divergence: if the observed patterns are caused by the effect of amino acid polymorphism reflecting mutation-selection biases they are expected to become less pronounced as divergence between genomes increases. Furthermore, there may be substantial differences in selection pressures on reciprocal amino acid substitutions [10]: changes from polar to non-polar amino acids in human proteins are more permissive than vice-versa. Such asymmetry and the degree to which is can contribute to the large-scale changes in amino acid composition has not yet been measured on the scale of several genomes.

Differences in patterns of selective pressure have also been predicted between evolutionary retained duplicated genes and single-copy genes [11-14]. Duplicated genes can persist in genomes either because one of the copies has acquired a new function (neofunctionalization [15-17]), or because both copies are now needed to perform the function or functions previously accomplished by a single copy (subfunctionalization). Subfunctionalizaton can occur either by means of partitioning of the ancestral functions between the two copies (for example by loss of one of alternative promoters in each copy), or by means of balanced degradation, i.e., fixation of hypomorphic alleles in each copy [18,19]. Each of these mechanisms implies relaxation of stabilizing selection, resulting in faster evolution in paralogs than in single-copy genes. Specifically, pure neofunctionalization occurs by accumulation of mutations in one of the copies, while the other remains under stabilizing selection [13,14,16,17]. Subfunctionalization occurring through balanced degradation, on the other hand, is accompanied by accumulation of deleterious mutations in both paralogs. Finally, subfunctionalization occurring by tissue- or developmental stage-specialization of gene expression without a change in functionality would result in retention of stabilizing selection action in both paralogs. It is much harder to make predictions about other types of subfunctionalization, such as subdivision of pre-existing multiple substrate specificity between duplicated genes, because the two functions may depend on different parts of coding portion of the gene and, therefore, retaining one but not the other may relax selective constraints acting on at least part of the sequence. Previous studies of duplicated genes in *Drosophila* genomes (e.g., [19]) detected elevated signal of positive selection in a subset of gene families with duplications using K_a_/K_s_ approach. Here we report a genome-wide analysis of differences between duplicated and single copy genes in frequency and spectrum of amino acid substitutions.

## Results

### Application of AcidMiner to *Drosophila* data: a database of amino acid substitutions in 12 genomes

The main purpose of AcidMiner is to extract amino acid substitutions data from multiple alignments and to expand them in the form of relational tables so then standard SQL can be used to perform queries by any combination of criteria and to calculate aggregates. AcidMiner takes raw data in the form of multiple alignments and Newick protein and species trees, processes it to produce derivative data such as parsimony-based polarization of substitutions and stores the result in a relational database structure. The raw data for the analysis reported here was a set of multiple amino acids alignments from 12 completely sequenced Drosophila genomes ([4,19]; see Methods). A set of SQL queries that can be run against this database to produce custom datasets with given restrictions and/or calculate any aggregates including statistical parameters on different datasets. In addition, for tasks not easily expressible in SQL, data already in the database to produce further derivative data. Examples of such tasks are: defining clades for each duplication, calculating number of substitutions in each clade (including cases when we can not unambiguously determine exactly which substitutions has occurred), calculating protein lengths in clades, calculating ages (timing data) of substitutions and duplications.

The resulting database in its current form includes 3,697,627 amino acid substitutions occurring in 12 drosophilid genomes spanning 11258 gene families. It consists of 14 tables defining the base data model. Two additional tables contain preloaded data for gene ontology and amino acid substitution properties, such as pair-wise change in polarity. Main tables include Families table, Tree Structure tables for protein and species trees with a separate record for each tree node and a branch terminating in this node, a Substitutions table with a record for each unambiguous and ambiguous substitution including a reference to branch where it occurred (or might have occurred for ambiguous substitutions) and a Duplications table, which includes phylogenetic information about each duplication and the two clades generated it. The database is available for download from AcidMiner website [20] in the form of a virtual machine. Any standard SQL tool can be used; queries for most of the queries we used for this study are also available in the AcidMiner repository, along with the source code and a detailed description of the database structure.

### Fluxes and asymmetries in amino acid substitutions

**Figure 1:**
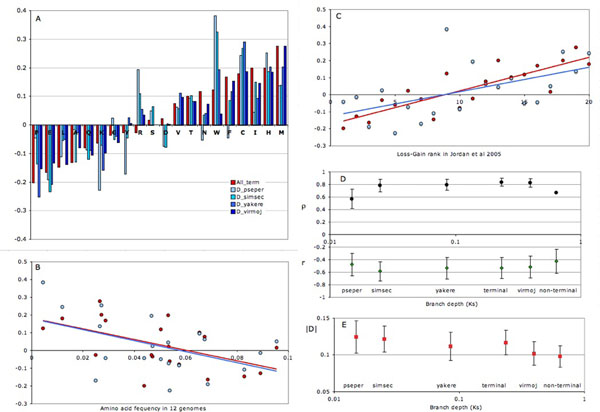
**Fluxes of amino acids in 12 *Drosophila* genomes**. A: Loser and gainer amino acids in the whole phylogeny (red bars) and terminal branches of different depth leading to sister species (blue bars; colour darkness increases with the depth of terminal branches). D_pseper – substitutions *in D. pseudoobscura* and *D. persimulans* branches; D_simsec – in *D. simulans* and *D. sechelia* branches; D_yakere – in *D. yakuba* and *D. erecta* branches; D_virmoj – in *D. virilis* and *D. mojavensis* branches. Relative amino acid gain D = (Gain-Loss)/(Gain+Loss) [3]. B: Relationship between relative amino acid gain (D) and frequency of each amino acid in 12 *Drosophila* genomes. Red circles – all 12 genomes, blue circles – only substitutions in the most shallow branches (*in D. pseudoobscura* and *D. persimulans*). C. Relationship between relative amino acid gain (D) and gain-loss rank in Ref [3]. Symbols as on Fig 1B. D. Rank (Spearman) correlation between relative amino acid gain (D) in branches of different depths in this study and in Ref [3] (ρ; black circles); Pearson coefficient of correlation between D and amino acid frequency in 12 *Drosophila* genomes (r, green diamonds). E. Mean pair-wise asymmetry of reciprocal substitutions (|D|, red squares). Branch depth (K_s_) on parts D and E is in synonymous substitutions per 4-fold degenerative site [4].

Figure 1 shows the results of amino acid fluxes analysis (data available in Additional file 1). As has been previously shown[3], frequent amino acids, in particular alanine, glutamic acid, leucine and proline, tend to be lost more often than created in protein sequences, while rare amino acids (in particular cysteine, histidine and methionine) are created more often than lost (Fig. 1 A, B ). There is a strong rank correlation between relative gain of amino acids in this study and in Ref [3], based on a variety of genome triplets, mostly prokaryotic (Fig. 1C). The general pattern of relative gain-loss is the same in the entire 12-genome phylogeny (Fig. 1A, red bars) and in pairs of sister species of different divergence depth (Fig. 1A, blue bars), however, there are exceptions. For example, phenylalanine and asparagines, which are moderate gainers in the entire phylogeny, show a net loss in the shallowest branch (*D. persimulans/D. pseudoobscura*), while arginine, a weak loser in the whole phylogeny shows a strong net gain in the shallow branches.

Contrary to the prediction based on the effect of intraspecific polymorphism [7,9], the observed gain-loss pattern does not become less pronounced as the divergence between genomes increases (Fig 1 D, E ; Additional file 2). Rank correlation with the global gain-loss pattern from Ref [3] slightly increases with branch depth, while mean pair-wise asymmetry (|D| calculated for each amino acid pair) and correlation with amino acid frequency remains flat. There is a slight tendency towards decrease of mean asymmetry (|D|) with the depth of phylogeny (Fig. 1 E), but neither of the pair-wise comparison of shallow vs. deeper branches is significant.

**Figure 2:**
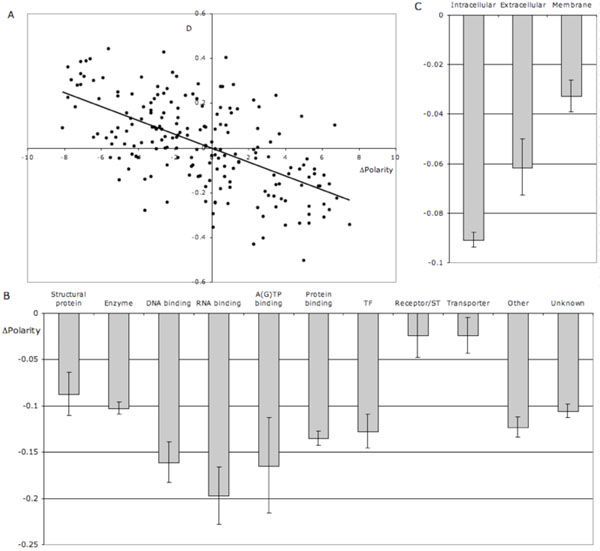
**Amino acid polarity and asymmetry of net gains and losses**. A. Correlation between relative net gain (D) and difference in polarity (Destination-Source) for 190 pair of amino acids. B. Net decrease of mean amino acid polarity due to substitutions in proteins of different molecular functions. C. Net decrease of mean amino acid polarity due to substitutions in proteins of different cellular localization.

Pair-wise asymmetry of amino acid gains and losses had a clear manifestation in terms of average change in amino acid polarity. Amino acid pairs with the largest polarity gain had the highest asymmetry towards net gain of the less polar amino acid (Fig. 2A). The degree of polarity asymmetry differed among genes of different functionality (Fig. 2B): nucleic acid- and nucleotide-binding proteins had the strongest asymmetry towards net gain of non-polar amino acids, while in receptor and transporter proteins such asymmetry was not observed. Likewise, net loss of polarity was the highest in proteins with intracellular localization, intermediate in proteins with extracellular localization and the lowest in membrane proteins, indicating the role of hydrophobicity of the protein’s cellular environment on relative gain and loss rate of polar and non-polar amino acids.

### Frequencies and radicality of amino acid substitutions in duplicated genes

**Figure 3:**
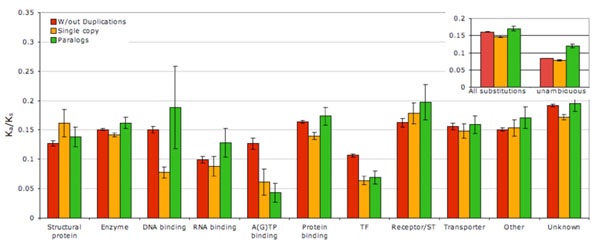
**Relative frequency of amino acid substitutions in single copy and duplicated genes**. K_a_ = Number of amino acid substitutions per amino acid site; K_s_ = cumulative number of synonymous substitutions per 4-fold degenerative site [4], i.e. cumulative length of branches leading to either single copy or duplicated genes. K_a_ / K_s_ for single copy and duplicated branches calculated for each gene family separately and averaged by molecular function without weighing. Standard errors shown reflect variance among gene families. Red bars: single copy genes in gene families without duplications; orange bars: single copy genes in gene families with duplication; green bars – duplicated genes. Inset: All substitutions and unambiguous substitutions for all gene families combined.

Duplicated genes appeared to accumulate more amino acid changes since duplication (per unit of time measured in units of synonymous substitutions per 4-fold degenerative site) than single copy genes (Fig. 3). Although the difference was statistically significant, it was not drastic: among 1701 gene families with duplications and with at least 1 substitution in both duplicated and unduplicated parts of the phylogeny paralogs accumulated more substitutions per unit of branch lengths than single copy genes in 988 families (58%; sign test P<0.00001). This relationship also varied across functional groups of genes, being the strongest in non-TF DNA-binding proteins, weaker in enzymes and protein-binding proteins and undetectable or reversed in other functional groups of proteins. Overall the rate of substitutions was the greatest in paralogs and the lowest in unduplicated sections of phylogenies of gene families with duplications, both when all substitutions and unambiguous substitutions only were considered (Fig. 3 inset).

**Figure 4:**
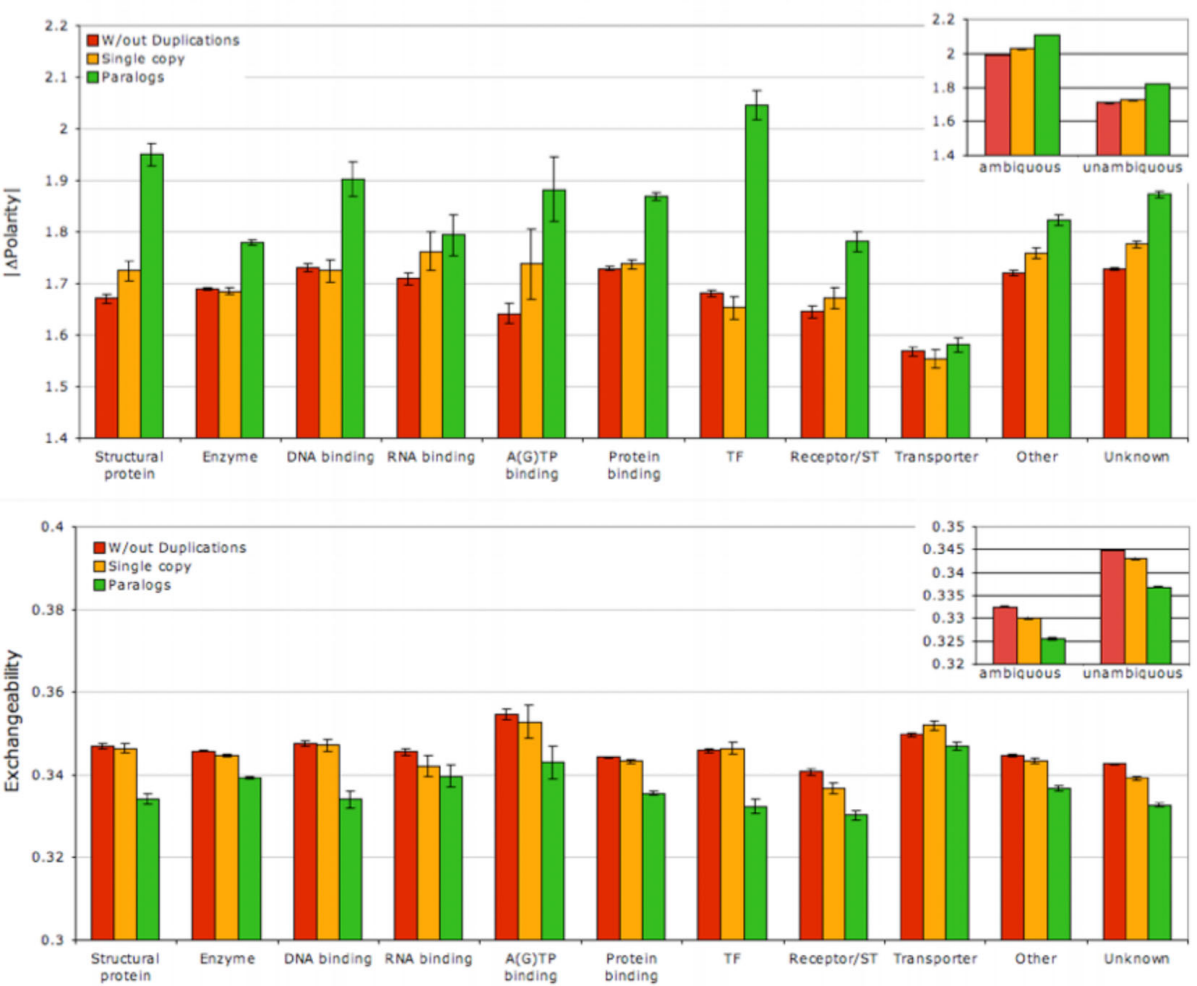
**Radicality of amino acid substitutions in single copy and duplicated genes.** A: mean absolute change of polarity between destination and source amino acids in gene families with different molecular function. B: mean exchangeability [21]. Colours as on Fig. 3. Insets: comparison of ambiguous and unambiguous substitutions.

Paralogs also evolved by more radical substitutions. Across functional groups of proteins (with the exception of transporter proteins) duplicated portions of phylogenies accumulated amino acid substitutions with greater average absolute change in polarity (Fig. 4A), while single copy genes typically did not differ significantly from gene families without duplications. Likewise, both overall and in every single functional category, paralogs differed by amino acid pairs with lower Exchangeability [21] (Fig. 4B). Again, single copy genes in families with duplications were intermediate between genes with no duplications and paralogs overall and typically did not differ from genes with no duplications within each functional category.

**Figure 5:**
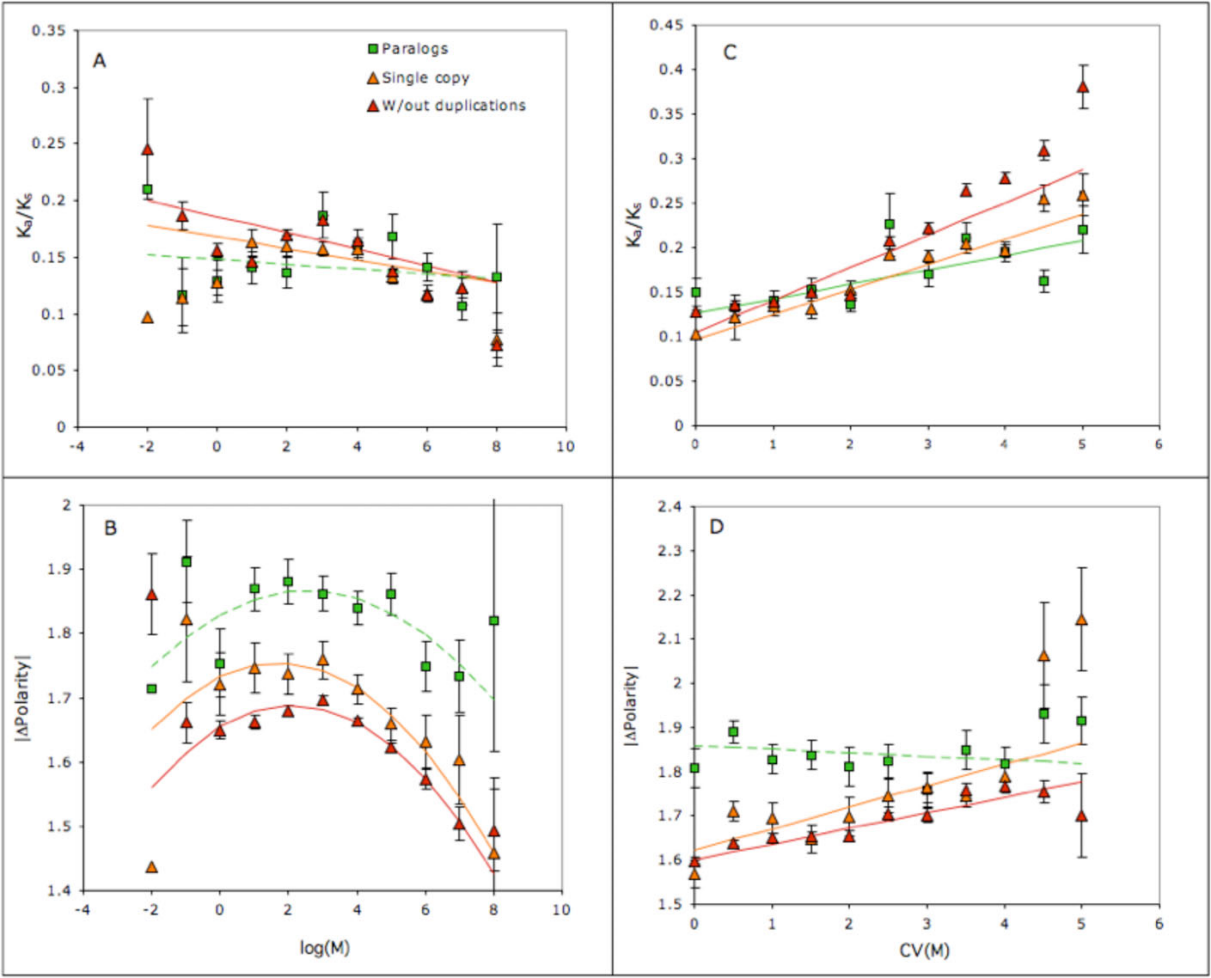
**Rates and radicality of amino acid substitution vs. expression level and ubiquity.** Relationship between relative substitution rate (K_a_/K_a_; A, C) and mean absolute change of polarity (|ΔP|; B, D) and log mean gene expression rate in whole fly (A, B) and coefficient of variation of expression rate across larval and adult tissues (C, D). Expression data from [22]. Solid lines: regressions significant at P<0.0001; dashed lines: regression without significant terms (shown for a comparison). Second-degree polynomial regression lines are shown when the quadratic term is significant, otherwise a linear regression is used.

As expected, substitution rates and radicalilty decreased with mean expression rate in the whole fly and increased with the coefficient of variance of expression rate across larval and adult tissues [22] (Fig. 5), corroborating previously observed patterns of stronger selective constraints in highly expressed genes and in household genes [23-25]. However, both effects were much less pronounced in paralogs than in single-copy genes; neither regression over mean expression level was significant (Fig. 5 A, B) and, while relative rate of substitutions increased with CV of expression rates across tissues, difference in polarity showed no correlation in paralogs. To summarize this pattern, the rate and radicality of duplicated genes evolution appeared to be uniformly high independently from gene expression rate and ubiquity. Data on rates and radicality of amino acid substitutions organized by gene family are available in Additional file 3.

### Clade asymmetries in duplicated genes

Table 1 summarizes the extent of asymmetry among clades resulting from duplication events. Substitution counts show a significant clade asymmetry in a large number of duplications. Asymmetry in radicality measures (|DPolarity| and Exchangeability) survives multiple tests correction in a lower number of tests. Total number of tests is different, because asymmetry was tested for all duplications, while other parameters – only for duplications, in which both clades had at least 2 unambiguous substitutions. Excluding terminal branches of the phylogeny, potentially contaminated by substitutions in pseudogenes and therefore biased towards clade asymmetry, does not change the result.

**Table 1:** Summary of clade asymmetries: the number of tests withstanding false discovery rate and Bonferroni adjustments for multiple tests. Tests: number of substitutions – χ^2^ test for heterogeneity; |DPolarity| and Exchangeability – t-test.

	All duplications			Terminal duplications excluded		
Asymmetry parameter	Number of duplications tested	FDR = 0.01	Bonferroni adjusted P = 0.01	Number of duplications tested	FDR = 0.01	Bonferroni adjusted P = 0.01
Total substitutions	4646	908	805	3118	804	741
Unambiguous substitutions	4646	721	621	3118	613	543
|DPolarity|	2964	66	39	2351	62	30
Exchangeability	2964	62	38	2351	58	30

**Figure 6:**
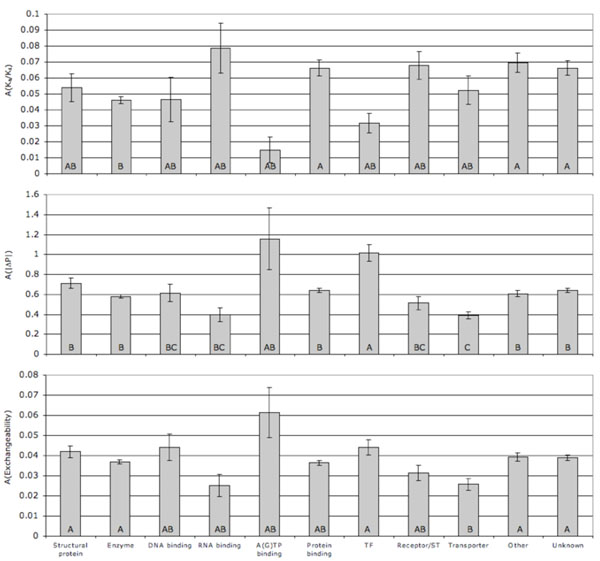
**Clade asymmetries in families with duplications.** Clade asymmetry (A) in relative substitution rate (top), absolute change in polarity (middle) and exchangeability (bottom) by molecular function. Molecular function category means were calculated by unweighted averaging over families. One-way ANOVA, respectively: F = 3.91, P < 0.00001; F = 7.63, P < 0.00001; F = 2.99, P < 0.001. Different letters signify categories different by Tukey-Kramer test, P = 0.05.

Clade asymmetries by molecular function categories are presented on Fig. 6. Protein- and RNA-binding proteins were characterized by the highest asymmetry of substitutions rates, while nucleotide-binding proteins and transcription factors had the lowest (although only enzymes vs. protein-binding proteins comparison is significant by Tukey-Kramer test). Nucleotide-binding proteins, on the other hand, demonstrated the highest asymmetry in both absolute polarity change and exchangeability of substitutions in the two clades, along with transcription factors, enzymes and structural proteins. The lowest radicality clade asymmetry was seen in RNA-binding and transporter proteins. Data on rates and radicality of amino acid substitutions organized by duplications are available in Additional file 4.

## Discussion

Several caveats in the data and analysis require attention. Firstly, alignments we used may contain pairs of paralogs, in which one of the copies is undergoing pseudogenization and is nor longer expressed, but has not yet acquired a frameshift, which would allow it to be recognized as a pseudogene. Indeed, there is a significant excess of nonsense mutations (per missense) present in the terminal branches of phylogeny (data not presented), indicating presence of pseudogenes in the alignments. Pairs of paralogs, in which one gene copy is undergoing pseudogenization, will demonstrate clade asymmetry, mimicking the signature of neofunctionalization. However, such paralogs are almost certainly present only in the most terminal branches of Drosophila phylogeny spanning over 70 mln years, because the half-life of duplications, in which one of the copies undergoes pseudogenization, is 2-4 mln years (12; 26). Terminal branches include a minority of duplications in our database and excluding such branches from the analysis does not alter the results (Table 1). This indicates that the observed clade asymmetry is not an artefact of pseudogenes. A direct comparison of clade asymmetries in terminal vs. non-terminal duplications is not possible for two reasons. Firstly, there are much fewer substitutions in the terminal branches, so there is an intrinsic difference in statistical power. Secondly, clade asymmetry analysis is based on unambiguous substitutions and the frequency of unambiguous substitutions increases with the depths of the phylogeny, possibly biasing such comparison.

On the other hand, some true functional paralogs may be missing from the alignments, particularly those resulting from ancient duplications, due to homology below the threshold used by the reciprocal BLAST algorithm (see Methods). This creates a bias towards less divergent paralogs, reducing our ability to detect elevated rates of evolution in duplicated genes. Relative magnitude of these opposing biases remains unknown.

Further, results presented in Table 1 do not necessarily indicate that clade asymmetries are more likely to manifest themselves in substitution rates than in substitution radicality. The number of test surviving multiple test correction probably reflects differences in statistical power rather than a true biological phenomenon.

Systematic loss/gain asymmetry in amino acid composition in 12 *Drosophila* genomes corroborates patterns previously observed in a variety of taxonomically diverse triplets of genomes [3]. This pattern does not become less pronounced as more and more distant genomes are included into consideration, indicating that it is not caused by the effect of polymorphisms reflecting mutation-selection balance influenced by mutational asymmetries [7,9].

We also demonstrate that this net loss/gain asymmetry is strongly correlated with source and destination amino acid polarities: substitutions of polar amino acids by non-polar ones have a higher net rate than the reciprocal substitutions. In the past we have demonstrated a similar polarity-related asymmetry in selection coefficients against amino acid substitutions in human proteins [10]; however this asymmetry was largely limited to strong selection (i.e., selection against clinically important phenotypes) and was not seen in evolutionary substitution rates.

One may hypothesise that replacing polar amino acids by any is less disruptive for the protein function because polar amino acids have a lower tendency to be located internally in the tertiary protein structure [21]. If so, we would expect the decrease of polarity due to amino acid substitutions to be the lowest in membrane proteins, in which polar amino acids in within-membrane domains tend to be internally located. Indeed, the decrease of polarity due to substitutions is the weakest in receptor and transporter proteins, many of which have membrane-embedded hydrophobic regions (Fig. 2 B) and in proteins with membrane localization (Fig. 2 C).

A question remains how is it possible that asymmetry in amino acid gains and losses systematically removed polar amino acids more often than non-polar ones (Fig.2A) over 70 mln years of drosophilid evolution (and actually over much longer period of evolution of proteins of much broader taxonomic spectrum [3])? There is no evidence that the relationship shown on Fig. 2A has a tendency to weaken in the most recent branches of the phylogeny (data not reported), which would have indicated an approach to an equilibrium. Rather, the frequencies of amino acids in proteins appear to be far from an equilibrium and we observe a constant turnover of polar amino acids due to more relaxed selective constraint acting on the amino acid of external location. One may further speculate that perhaps such systematic loss of surface polar amino acids would gradually change protein folding as external sites become occupied by more hydrophobic amino acid residuals. This process may be a potentially important mechanism of acquiring new functions by duplicated genes.

We have demonstrated that, in a genome-wide assessment, duplicated genes evolve both faster (higher K_a_/K_s_) and through more radical amino acid substitutions (higher |DPolarity|, lower exchangeability) than single copy genes (Figs 3 and 4). Likewise, single copy genes in families with extant duplications tend to evolve faster and more radically than single copy genes in families without extant duplications, indicating that duplications are more likely to be retained in gene families with weaker selective constraints.

Just like with the signed polarity change, the absolute change of polarity is not significantly different between duplicated and single copy genes among genes coding for transporter proteins, corroborating the hypothesis of the importance of relaxed selective constraint on surface sites of water-soluble proteins (Fig. 4 A). (This difference is, however, significant for receptor proteins.) The exchangeability index, on the other hand, is significantly lower in duplicated transporter proteins, suggesting that paralogs in these genes families do evolve through more radical substitutions, just without systematic net loss of polar residuals.

Data on the asymmetry of clades resulting from duplications supports the hypothesis of widespread neofunctionalization accompanying retention of duplicated genes: over 1/3 of all duplications show a significant asymmetry in amino acid substitution rates with false discovery rate 0.05 and almost 1/5 of all substitution show asymmetry, which stands Bonferroni correction (Table 1). Much fewer duplications show a significant asymmetry in radicality of substitutions, although about 6% have a significant asymmetry in absolute polarity change (with false discovery rate 0.05). Gene families of different functionality differ from each other in the degree of clade asymmetry with a hint of a negative correlation between asymmetry in rates (Fig. 6, top) and asymmetry in radicality (Fig. 6, middle and bottom). No molecular function category stands out in terms of tendency to display signatures of neofunctionalization, although RNA-binding proteins have the lowest (non significant) difference in rates and radicality of substitutions between duplicated and single copy genes (Fig. 3 and 4) and the lowest clade asymmetry of substitution radicality in paralogs (Fig. 6), indicating that, perhaps, in these proteins neofunctionalization is less common. Interestingly, transcription factors appear to show low neofunctionalization signal in terms of substitution rates (no difference between duplicated and single-copy genes, Fig. 3; low asymmetry between paralogs, Fig 6, top), but a strong neofunctionalization signal in terms of substitution radicality (Fig 4; Fig. 6 middle and bottom). One may hypothesize that positive selection for a novel functionality can operate either by increased rate of substitutions, or by favouring more radical changes without the increase of rates.

## Conclusions

We have designed a tool, which allows a detailed phylogenetic analysis of amino acid substitutions in a large number of multiple alignments with or without duplicated genes present. The algorithm is capable to polarize and establish phylogenetic position of all substitutions for which it is possible (unambiguous) and to list all possible alternatives for other, ambiguous substitutions. It results in a database, which can be used to answer questions about patterns of amino acids substitutions genome-wide or in particular categories of genes such as molecular functions or duplication status.

The analysis of such database of substitutions in 12 Drosophila genomes confirmed previously observed non-equilibrium patterns of net losses and gains of individual amino acids, demonstrated that these patterns do not weaken with the depth of phylogeny and revealed a strong correlation between polarity of amino acid and propensity to display a net loss. We hypothesize that this effect can be explained by relaxed selective constraints on externally located amino acid sites occupied by polar residuals. Evolution of duplicated genes is characterized by both higher relative rate of substitution and more radical nature of these substitutions, as compared to single copy genes. The rate and radicality in paralogs displays a weaker relation with mean expression rate and variance of expression rates across tissues than in single copy genes. This pattern, along with the strong asymmetry between clades resulting from duplication events, indicates widespread neofunctionalization of retained duplications.

## Methods

### Algorithm, data provenance and phylogenetic analysis

A new a phylogenetic analysis tool AcidMiner [20] is used to convert raw data in the form of protein alignments and Newick protein and species trees into a relational database of amino acid substitutions searchable by standard SQL queries and containing a number of preset queries. Additionally, it allows further derivative data to be produced for tasks not easily expressible in SQL. Code for such purposes can be written either in Java or as stored procedures in the DBMS proprietary language, which in some cases results in faster processing. AcidMiner Java code, custom DBMS procedures and most of the complex SQL queries used in this study are also available [20].

Protein alignments and corresponding phylogenies were acquired from Dfam database at Indiana University [19,27]. These alignments have been obtained by means of modified reciprocal BLAST method [4,19]. Briefly (see [19] for details), the results of an all-by-all comparison between the 12 genomes using BLASTP are filtered to retain as homologs all hits with E-values within two orders of magnitude of the highest hit. Gene families (clusters of homologs) are then deterimined by finding the maximally connected clusters that are disjoint from one another while discarding nonreciprocal relationships [19].

NOTUNG phylogenies reconciling topological incongruence between species trees and proteins trees [28] were used to map duplications and substitutions. We considered 11258 gene families (with at least 6 species represented), which contained 8,766,256 amino acid sites. Areas of alignments with >1 indels in a row in one or more species were excluded from the analysis. Of the amino acid sites retained for the analysis 2,131,864 sites had at least one substitution in at least one clade. These sites contained a total of 3,697,627 substitutions. A substitution was called unambiguous if it could be unequivocally polarized and placed on the phylogeny by the genotype of the outgroup clade; there were 2,004,536 such substitutions. Substitutions without a single most parsimonious placement were called ambiguous; such substitutions were included into the rates calculated, but excluded from the analysis of radicality of substitutions. Substitution data arranged by amino acids, by gene families and by duplications are available in supplemental materials or by request.

Paralogs were identified as homologs present in the same genome and substitutions were considered to have been acquired by duplicated genes if their most parsimonious placement on the phylogeny is more terminal than the placement of the duplication event. Conversely, substitutions occurring in branches basal to the most ancient surviving duplication in a clade were considered to have occurred in a single-copy gene.

### Fluxes, asymmetries, radicality and substitutions rates

Net relative gain (or loss) of amino acids through substitutions (flux) was characterized by the parameter D = (C-R)/(C+R), where C is the number of times each amino acids was created and R – the number of times the same amino acid has been removed by substitutions [3]. The parameter D was be calculated separately for each amino acid pair, or for each amino acid as a marginal value. Change of amino acid polarity due to substitutions was calculated as mean difference between source and destination amino acid polarities (Polarity values taken from AAIndex, Ref. [29]). The absolute value of this difference, |DPolarity|, was used as a measure of radicality of each amino acid substitution; an alternative, inverse measure of radicality used was the Exchangeability index [21].

Each gene family was characterized by a K_a_/K_s_ value, obtained in the following manner. K_a_ was estimated as the ratio of the number of substitutions (in either the whole tree, or separately for duplicated and unduplicated portions of the tree) to the number of amino acid sites in the alignment. K_s_ was calculated as the sum of branch lengths of the corresponding section of the tree expressed as the frequency of synonymous substitutions per 4-fold degenerative site [4].

### Ontology and expression data and statistical analysis

Gene ontology and gene expression data were merged with amino acid substitution data by *D. melanogaster* genes FlyBase IDs [30]. Therefore, for all analyses involving molecular functions and gene expression level, genes families lacking a *D. melanogaster* gene were excluded. Conversely, families with duplicated *D. melanogaster* genes appeared in these types of analysis with the number of times equal to the number of *D. melanogaster* paralogs they contained. Gene families were subdivided into the following molecular function categories using FlyBase ontology data [30]: structural proteins, enzymes, transcription factors, other DNA-binding proteins, RNA-binding proteins, ATP- and GTP-binding proteins, receptors and signal transduction proteins, transporters, proteins with other functions and proteins with unknown function. Gene expression data were obtained from FlyAtlas database [22].

## Authors' contributions

LYY proposed the study methodology, accomplished data analysis and prepared the manuscript. MAB wrote software, generated the dataset and contributed to the manuscript preparation.

## Competing interests

The authors declare that they have no competing interests.

## Supplementary Material

Additional file 1 Excel spreadsheet with pair-wise amino acid substitution frequencies mapped to terminal branches of the phylogeny, by species. Click here for file

Additional file 2 Excel spreadsheet with pair-wise amino acid substitution frequencies, separately for terminal and non-terminal branches. Click here for file

Additional file 3 Excel spreadsheet with data on rates and radicalities of substitutions by gene family. Click here for file

Additional file 4 Excel spreadsheet with data on rates and radicalities of substitutions by duplication with separate columns for each of the two clades resulting from each duplication events. Click here for file
